# Antidepressant prescription patterns and CNS polypharmacy with antidepressants among children, adolescents, and young adults: a population-based study in Sweden

**DOI:** 10.1007/s00787-018-01269-2

**Published:** 2019-01-19

**Authors:** Tyra Lagerberg, Y. Molero, B. M. D’Onofrio, L. Fernández de la Cruz, P. Lichtenstein, D. Mataix-Cols, C. Rück, C. Hellner, Z. Chang

**Affiliations:** 1 Department of Medical Epidemiology and Biostatistics, Karolinska Institutet, Nobels väg 12A, Stockholm, Sweden; 2 Department of Psychiatry, Warneford Hospital, University of Oxford, Oxford, UK; 3 Department of Clinical Neuroscience, Karolinska Institutet, Solna, 171 65 Stockholm, Sweden; 4 Department of Psychological and Brain Sciences, Indiana University, Bloomington, IN USA; 5 Stockholm Health Care Services, Stockholm County Council, Stockholm, Sweden

**Keywords:** Antidepressants, Polypharmacy, Child and adolescent, Young adults, Register, Sweden

## Abstract

This study examines trends in antidepressant drug dispensations among young people aged 0–24 years in Sweden during the period 2006–2013, as well as prescription patterns and central nervous system (CNS) polypharmacy with antidepressants. Using linkage of Swedish national registers, we identified all Swedish residents aged 0–24 years that collected at least one antidepressant prescription (here defined as antidepressant users) between 1 January 2006 and 31 December 2013 (*n* = 174,237), and categorized them as children (0–11 years), adolescents (12–17 years), and young adults (18–24 years). Prevalence of antidepressant dispensation rose from 1.4 to 2.1% between 2006 and 2013, with the greatest relative increase in adolescents [by 97.8% in males (from 0.6 to 1.3%) and by 86.3% in females (from 1.1 to 2.1%)]. Most individuals across age categories were prescribed selective serotonin reuptake inhibitors, received their prescriptions from psychiatric specialist care, and had treatment periods of over 12 months. Prevalence of CNS polypharmacy (dispensation of other CNS drug classes in addition to antidepressants) increased across age categories, with an overall increase in prevalence from 52.4% in 2006 to 62.1% in 2013. Children experienced the largest increase in polypharmacy of three or more psychotropic drug classes (4.4–10.1%). Anxiolytics, hypnotics, and sedatives comprised the most common additional CNS drug class among persons who were prescribed antidepressants. These findings show that the dispensation of antidepressants among the young is prevalent and growing in Sweden. The substantial degree of CNS polypharmacy in young patients receiving antidepressants requires careful monitoring and further research into potential benefits and harms.

**Electronic supplementary material:**

The online version of this article (10.1007/s00787-018-01269-2) contains supplementary material, which is available to authorized users.

## Introduction

Mood and anxiety disorders are common mental problems in young individuals [1, 2] and may contribute to both short- and long-term adverse outcomes—including decreased quality of life, lower school performance, and suicide in severe cases [3–6]. Antidepressants are the main pharmacological treatment option available and have become increasingly common in several Western settings [7–10]. For example, the prevalence of receiving antidepressant prescriptions in those aged 0–19 years rose by 54.4% in the UK (from 0.7 to 1.1%) and by 49.2% in Germany (from 0.3 to 0.5%), during 2005–2012 [8]. Despite this, safety concerns about antidepressant use in this age group have been raised—with reports on increased risks of adverse outcomes such as violence [11], akathisia [12], and suicidality [13–15]. In 2004, the US Food and Drug Administration (FDA) issued a “black box” warning to highlight potential risks of antidepressants in individuals aged up to 18 years. In 2007, this warning was extended to those aged up to 25 years [14, 16]. Similar warnings were issued across Europe in the same period—for example, by the European Medicines Agency in 2005 [8].

In light of these safety concerns, it is important to monitor the risks and benefits of antidepressant use among the young. This entails documenting changes in the prevalence of dispensation, as well as clinical prescription patterns—including the source of prescription, treatment duration, and use of other central nervous system (CNS) drugs. CNS polypharmacy is of particular importance, as the safety and efficacy of combining psychotropic drug classes are not well established [17], with adverse effects including cumulative toxicity and drug interactions [18]. A US population-based study found that psychotropic polypharmacy of two drugs was associated with a 17% higher average number of side effects, while polypharmacy with three or more drugs was associated with a 38% higher average number, compared with monopharmacy [19]. Adverse outcomes resulting from polypharmacy may be compounded among the young, which comprise a potentially vulnerable population where the effects of individual drugs, let alone combinations of them, are not conclusively established. Many psychotropic drugs are also prescribed off-label in this age group—40.9% of all antidepressant prescriptions in German 0- to 17-year-olds were estimated to be off-label in 2011 [20]. The prevalence of CNS polypharmacy generally is understudied, although there is evidence that it has increased in certain settings. For example, among US outpatient visits where a psychiatric diagnosis was made, the percentage with prescriptions of psychotropic medications from two or more drug classes increased from 22.2% in 1996–1999 to 32.2% in 2004–2007 [21]. Recent studies on CNS polypharmacy among young antidepressant users in a European population are lacking.

Documenting the clinical patterns of antidepressant dispensations among the young has implications for the development of appropriate treatment guidelines, as well as for future research on the benefits and harms of antidepressant drugs in a potentially vulnerable age group. The present study therefore examines antidepressant dispensations among children, adolescents, and young adults in Sweden between 2006 and 2013—investigating patterns of type of antidepressant medication, source of prescription, treatment duration, and CNS polypharmacy.

## Methods

### National registers and study population

We linked information from three Swedish national registers based on unique personal identification numbers [22]. The first was the Swedish Prescribed Drug Register (PDR), which covers data on all dispensed pharmaceuticals in Sweden since July 2005, including drug identity [registered using Anatomical Therapeutic Chemical (ATC) codes], dose, dates of dispensed prescriptions, and the prescriber’s profession and practice [23]. The proportion of invalid dispensation entries in the PDR is expected to be below 2% [24]. The second was the National Patient Register, which contains information on all admissions to inpatient hospital care since 1973 and admission to outpatient specialists since 2001 [25], with information on all discharge diagnoses in accordance with International Classification of Disease (ICD) codes. Diagnosis data from the National Patient Register have good to excellent validity for a range of conditions, including depression, bipolar disorder, and schizophrenia [26–28]. The third was the Total Population Register, which includes demographic information on all Swedish residents since 1968, and has virtually complete coverage of births and deaths [29].

The target population included all individuals aged up to 25 years who were residing in Sweden at some point during the period between 1 January 2006 and 31 December 2013. The period from 1 July to 31 December 2005 was not used to allow for complete yearly data.

Within the target population, we selected all individuals who had at least one dispensed prescription of antidepressant drugs (ATC code: N06A) during the study period. We also created a 1:1 randomly selected control group of antidepressant non-users from the Total Population Register by matching the antidepressant users by year of birth and sex, to compare how psychiatric comorbidity profiles of antidepressant users differed from those of non-users between age groups.

### Measures

Antidepressant use was defined as dispensation of antidepressant drugs (ATC code: N06A) from the PDR. The antidepressants were grouped as: tricyclic antidepressants (TCAs; N06AA); selective serotonin reuptake inhibitors (SSRIs; N06AB); serotonin–norepinephrine reuptake inhibitors (SNRIs; N06AX16 or N06AX21); monoamine oxidase inhibitors (MAOI; N06AF or N06AG); and others (other N06A drugs) (Supplementary Table 1). From the PDR, we also retrieved information on the source of prescription (primary care/non-psychiatric specialist care/psychiatric care) and the duration of prescription [single dispensation, short term (≤ 6 months), medium term (6–12 months), and long term (> 12 months)] (24). We considered an individual to be receiving treatment during the interval between two dispensed antidepressant prescriptions, unless dispensed prescriptions occurred more than 6 months apart (25). If multiple treatment periods occurred during a year, the longest treatment period for each individual was considered.

Use of CNS drugs concurrently with an antidepressant prescription was defined as a dispensed prescription of at least one other CNS medication within 6 months of a dispensed antidepressant prescription. The other CNS drugs considered were: antipsychotics (N05A); anxiolytics, hypnotics, and sedatives (N05B or N05C); attention-deficit/hyperactivity disorder (ADHD) medication (N06B); drugs used in addictive disorders (N07B); opioids and pain medications (N02A); and antiepileptic drugs (N03A).

### Statistical analyses

We described trends in antidepressant dispensation by calculating the prevalence of dispensation by year for the period 2006–2013, overall and stratified by sex and age group [“children” (0–11 years), “adolescents” (12–17 years), and “young adults” (18–24 years)]. A Cochran–Armitage test for trend was performed. Antidepressant dispensation patterns for the year 2013 were presented in relation to antidepressant type, source of prescription (primary care/non-psychiatric specialist care/psychiatric care), and duration of prescription.

Central nervous system polypharmacy is here defined as a dispensed prescription of one or more other CNS drugs within 6 months of the dispensed antidepressant prescription [30]. The degree of CNS polypharmacy among patients receiving antidepressants over the study period was assessed by calculating the proportion of antidepressant users with different numbers of classes of other CNS drugs (0, 1, 2 or ≥ 3) from 2006 to 2013, stratified by age group.

We further examined the proportion of individuals collecting other CNS drugs among patients receiving antidepressants in the year 2013. Odds ratios (ORs) for the collection of other CNS drugs in those with dispensed antidepressant prescriptions versus controls were calculated by age group, using logistic regression models with adjustment for sex and age as a categorical variable (in 1-year bands) and presented alongside 95% confidence intervals (CIs) [31]. We also calculated the frequency of psychiatric disorders in individuals with dispensed antidepressants for the year 2013, alongside ORs for a given disorder in individuals dispensing antidepressants versus non-users. Psychiatric disorders were defined as diagnoses made prior to or during the year of the dispensed antidepressant prescription, including depression (ICD-10 codes: F32–F39), bipolar disorder (F30–F31), anxiety disorder (F40–F48), schizophrenia spectrum disorder (F20–F29), substance use disorder (F10–F19), personality disorder (F60–F61), ADHD (F90), and other developmental or childhood disorders (F80–F98, excluding F90).

All statistical analyses were performed using SAS version 9.4 (SAS Institute). Figures were created in R version 3.5.0.

## Results

### Time trends in antidepressant use

We identified 174,237 individuals resident in Sweden who received at least one prescription at any age below 25 years during 2006–2013. Table 1 shows the prevalence of antidepressant dispensation by year as stratified by age and sex. For the overall population, there was an increase in antidepressant dispensation, from 1.4% in 2006 to 2.1% in 2013 (Table 1). The greatest relative change in prevalence of antidepressant dispensation was in adolescents (by 97.8% in males, from 0.6 to 1.3%, and by 86.3% in females, from 1.1 to 2.1%). However, young adults (18–24 years) had the greatest prevalence overall (3.6% for males and 7.0% for females in 2013). With regard to the type of antidepressant, the greatest relative change in prevalence was in the dispensation of other antidepressants (by 89.3%, from 0.2 to 0.4%), followed by SNRIs (80.5%, from 0.1 to 0.2%) and TCAs (52.5%, from 0.06 to 0.10%; Supplementary Table 2). The most common antidepressant in 2013 across age groups was sertraline (accounting for 62.8, 29.0, and 28.6% of dispensed prescriptions in children, adolescents, and young adults, respectively), followed by fluoxetine in the youngest two age categories, but by citalopram in the young adults (Supplementary Table 3). Sertraline, fluoxetine, amitriptyline, and fluvoxamine are the only antidepressant types out of the ten most commonly dispensed ones in children and adolescents that have indications in these age categories (Supplementary Table 6). In children, 10.0% of the dispensed prescriptions pertained to drugs that are off-label in that age category, while the figure was 18.9% in adolescents in 2013 (Supplementary Table 3).

**Table 1:** Prevalence of antidepressant use in Sweden from 2006 to 2013

	2006	2007	2008	2009	2010	2011	2012	2013	Relative Change (%)^a^	*P* ^b^
All										
Total (%)	1.4	1.4	1.5	1.6	1.7	1.8	1.9	2.1	52.9	<0.001
Male (%)	0.9	1.0	1.0	1.1	1.2	1.3	1.4	1.5	60.8	<0.001
Female (%)	1.8	1.9	2.0	2.1	2.2	2.3	2.5	2.7	48.9	<0.001
Children (0–11 years)										
Male (%)	0.05	0.04	0.04	0.05	0.06	0.06	0.08	0.08	78.5	<0.001
Female (%)	0.02	0.02	0.02	0.02	0.03	0.03	0.04	0.04	52.9	<0.001
Adolescents (12–17 years)										
Male (%)	0.6	0.7	0.8	0.8	0.9	1.1	1.1	1.3	97.8	<0.001
Female (%)	1.1	1.3	1.3	1.4	1.5	1.7	1.9	2.1	86.3	<0.001
Young adults (18–24 years)										
Male (%)	2.5	2.6	2.7	2.8	2.9	3.2	3.4	3.6	46.3	<0.001
Female (%)	5.2	5.4	5.4	5.5	5.7	6.0	6.5	7.0	35.4	<0.001

All individuals aged below 25 years are included

^a^Percentage difference in year 2013 compared with year 2006

^b^Cochran–Armitage trend test

### Patterns of antidepressant prescription

Table 2 shows the patterns of dispensed antidepressant prescriptions in 2013 by type of antidepressant, source of prescription, and duration of treatment, stratified by sex and age group. Overall, there were similar patterns of use between the sexes. For both males and females, SSRIs were the single most prevalent antidepressant type. Other antidepressants were the second most common antidepressant type across ages, except among 0- to 11-year-old females, where TCAs held this spot. Mirtazapine and bupropion were common “other” antidepressants, accounting for 8.7% and 1.7% of all dispensed antidepressant prescriptions. The proportion of antidepressant users with a dispensed prescription for an SNRI or other antidepressant was the highest for young adults. Finally, 13.5% (*n* = 7969) of antidepressant users across age groups collected more than one type of antidepressant drug in 2013.

**Table 2:** Patterns of antidepressant use in 2013, by age group and sex

	Children (0–11 years)	Adolescents (12–17 years)	Young adults (18–24 years)
Male			
Number of users	583	3920	16,937
Type (%)^a^			
TCA	1.2	2.2	4.6
SSRI	95.9	92.7	75.0
SNRI	<0.1	1.5	13.3
MAOI	<0.1	<0.1	0.1
Other	3.6	8.6	25.2
Source (%)			
Primary care	2.7	8.3	41.3
Non-psychiatric specialist care	18.9	13.5	6.1
Psychiatric care	78.4	78.2	52.7
Duration of medication (%)			
Single prescription^b^	9.4	9.5	17.2
Short term (≤ 6 m)	9.6	10.3	13.2
Medium term (6 < *x* ≤ 12 m)	11.5	15.2	15.9
Long term (> 12 m)	69.5	65.0	53.7
Female			
Number of users	250	6113	31,051
Type (%)^a^			
TCA	6.4	2.7	5.5
SSRI	93.2	94.3	81.5
SNRI	<0.1	1.8	13.1
MAOI	<0.1	<0.1	0.1
Other	0.8	7.8	16.2
Source (%)			
Primary care	4.8	10.5	45.5
Non-psychiatric specialist care	21.2	7.2	6.3
Psychiatric care	74.0	82.3	48.2
Duration of medication (%)			
Single prescription**	9.2	7.8	13.9
Short term (≤ 6 m)	8.0	11.6	10.8
Medium term (6 < *x* ≤ 12 m)	12.0	14.8	15.5
Long term (> 12 m)	70.8	65.9	59.8

^a^ Tricyclic antidepressants (TCA); selective serotonin reuptake inhibitors (SSRI); serotonin–norepinephrine reuptake inhibitors (SNRI); monoamine oxidase inhibitors (MAOI)

^b^ Note that single prescriptions here entail single dispensed prescriptions of any antidepressant drug. “Switching” between different types of antidepressant drugs is not captured by this number

A majority of individuals dispensing antidepressant prescriptions in the 0- to 11-year and 12- to 17-year age groups received their prescriptions from psychiatric care (above 70%), with non-psychiatric specialist care as the second most common source except in female adolescents. In young adults, 52.7% of males and 48.2% of females received their prescriptions from psychiatric care. For all age groups and for both sexes, the majority of antidepressant users had treatment periods longer than 1 year.

### Central nervous system polypharmacy

**Fig. 1:**
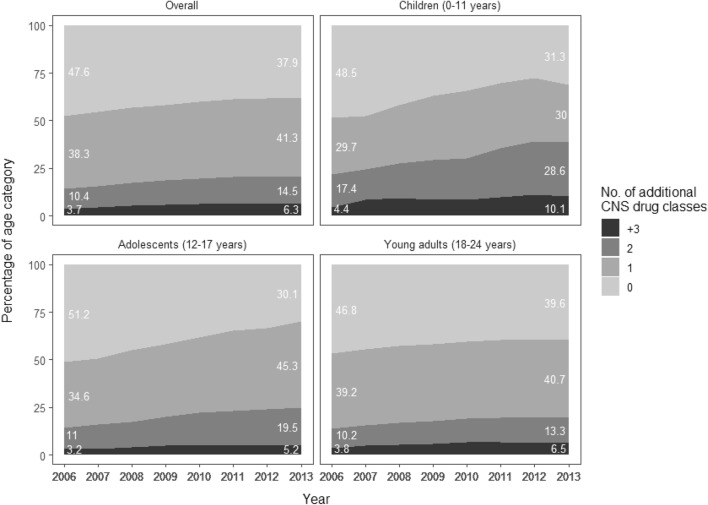
Percentage of antidepressant users with different levels of CNS polypharmacy 2006–2013

Figure 1 presents the changes in CNS polypharmacy among antidepressant users of different ages during the period 2006–2013. CNS polypharmacy of at least one additional CNS drug class increased in all age categories over the study period (from 52.4 to 62.1%)—with the biggest increase (48.8–69.9%) in the adolescent category. However, the largest increase in polypharmacy of two and three or more psychotropic drugs in addition to antidepressants was in children (from 17.4 to 28.6% and from 4.4 to 10.1%, respectively). This age category also had the highest proportion of antidepressant users with dispensed prescriptions of three or more psychotropic drugs in addition to antidepressants throughout the period.

Table 3 shows the proportion of antidepressant users who collected additional CNS drugs, alongside the ORs for receiving other CNS drugs in antidepressant users versus controls. In all age groups, anxiolytics, hypnotics, and sedatives were the most common class of drugs, with prescriptions collected by 49.8, 57.1, and 53.9% of antidepressant users in the child, adolescent, and adult populations, respectively. In the two youngest age categories, this was followed by ADHD medication (47.7% prevalence in children and 27.5% in adolescents) and antipsychotics (19.0% and 13.0%, respectively). In the 18- to 24-year-olds, antipsychotics was the second largest group (13.2%), followed by opioids (12.6%). Individuals collecting antidepressant prescriptions were more likely to use other CNS drugs than population controls. Anxiolytics, hypnotics, and sedatives had the highest OR of being dispensed in antidepressant users versus controls in all age groups [OR of 64.1 (95% CI 36.4–112.7) in children, 68.0 (95% CI 58.6–78.7) in adolescents, and 44.4 (95% CI 41.8–47.1) in young adults]. This was followed by antipsychotics for all age groups.

**Table 3:** Dispensation of common central nervous system (CNS) medications among individuals using antidepressants in 2013, by age group

	Anti-psychotics	Anxiolytics, hypnotics, and sedatives	Attention-deficit/hyperactivity disorder medication	Drugs used in addictive disorders	Opioids	Anti-epileptics
Children (0–11 years)						
User (*N* = 833)	158 (19.0%)	415 (49.8%)	397 (47.7%)	1 (0.1%)	14 (1.7%)	50 (6.0%)
Control (*N* = 833)	4 (0.5%)	13 (1.6%)	25 (3.0%)	0 (0.0%)	0 (0.0%)	5 (0.6%)
OR (95% CI)^a^	52.0 (18.8, 142.7)	64.1 (36.4, 112.7)	31.6 (20.7, 48.4)	–	–	10.7 (4.2, 27.0)
Adolescents (12–17 years)						
User (*N* = 10,033)	1300 (13.0%)	5725 (57.1%)	2756 (27.5%)	33 (0.3%)	486 (4.8%)	531 (5.3%)
Control (*N* = 10,033)	23 (0.2%)	195 (1.9%)	271 (2.7%)	2 (0.0%)	124 (1.2%)	51 (0.5%)
OR (95% CI)^a^	65.2 (43.1, 98.6)	68.0 (58.6, 78.7)	14.4 (12.6, 16.4)	16.6 (4.0, 69.0)	4.1 (3.4, 5.0)	11.0 (8.2, 14.6)
Young adults (18–24 years)						
User (*N* = 47,988)	6337 (13.2%)	25,848 (53.9%)	5219 (10.9%)	947 (2.0%)	6050 (12.6%)	5258 (11.0%)
Control (*N* = 47,988)	192 (0.4%)	1233 (2.6%)	550 (1.1%)	45 (0.1%)	1642 (3.4%)	414 (0.9%)
OR (95% CI)^a^	38.1 (33.0, 44.0)	44.4 (41.8, 47.1)	10.7 (9.8, 11.7)	21.5 (15.9, 29.0)	4.1 (3.9, 4.3)	14.2 (12.8, 15.7)

^a^Adjusted for sex and age (in 1-year bands)

Finally, antidepressant users presented higher proportions of psychiatric disorders as compared to population controls (Supplementary Table 5). Out of all the common psychiatric disorders considered, child antidepressant users had the highest ORs of having an anxiety disorder diagnosis, as compared to population controls. For adolescents and young adults, antidepressant users had the highest ORs of a depression diagnosis, compared to population controls.

## Discussion

In this population-based study of antidepressant dispensations among young individuals in Sweden, we found a number of noteworthy patterns over the study period (2006–2013). First, the dispensation of antidepressant drugs increased across all age categories, with the greatest relative increase in adolescents. Second, most individuals collected prescriptions for SSRIs, received their prescriptions from psychiatric specialist care, and had treatment periods of over 12 months. Third, there was extensive co-medication with other CNS drugs; the proportion of individuals collecting antidepressants at the pharmacy with any level of CNS polypharmacy rose from 52.4% in 2006 to 62.1% in 2013. To our knowledge, this is the first nationwide study showing the growth in CNS polypharmacy over this period and in this age group.

The prevalence of antidepressant dispensation increased in all age categories over the study period, with the 12- to 17-year age group seeing the greatest percentage rise (by 97.8% in males and 86.3% in females). A similar rise in antidepressant use has been documented among the young in many Western settings over the last decade—including in Dutch, British, American, Danish, and German populations over the period 2005–2012 [8]. By contrast, in a French study, the prevalence of antidepressant use rose only marginally among those aged 6–17 years (from 0.51 to 0.53%), and fell in the 6- to 11-year-olds, during 2009–2016 [32]. On the one hand, the increase documented in our study may be warranted by higher rates of diagnosed mood and anxiety disorders in the population. For example, a US-based study found that major depressive episodes among adolescents and young adults increased over the period 2005–2014 [7]; and is the source for the evidence presented in the sentence “evidence from Sweden indicates that the proportion of individuals aged 16–24 who self-reported mental ill-health more than tripled between 1988–1989 and 2004–2005 [33]; and that there was a marked increase in the proportion of individuals seeking care for psychiatric problems among the young in 2000–2014 in Stockholm [34]. On the other hand, there is a debate about whether antidepressants are efficacious treatment alternatives in this age group, and whether they have acceptable trade-offs between benefits and harms [35]. In particular, studies suggest an increased risk of suicidality and aggression in antidepressant users under age 25 years [13, 36–38], meaning that the rise of antidepressant dispensation in this age group should be monitored closely. Consistent with findings that the onset of most major mood and anxiety disorders occurs in adolescence or early adulthood [39], there was a higher prevalence of having dispensed one or more antidepressant prescriptions with increasing age over the study period. We also observed that antidepressant use was about two times more prevalent among women compared to men in adolescents and young adults, which is a pattern found among adults in many countries [40–45], as well as among the young in other European populations [32]. However, males had higher prevalence of antidepressant use than females in the 0- to 11-year-olds in our study, which warrants further investigation.

SSRIs constituted the most common antidepressant drug class across all age categories. This is in accordance with the current guidelines from the Swedish National Board of Health and Welfare, where SSRIs are recommended as the first-line pharmacological treatment for mood and anxiety disorders across ages [46]. However, SSRIs were somewhat less common in young adults compared to the younger age groups. This may be the result of SSRIs being the recommended first-line pharmacological treatment for mood and anxiety disorders among children and adolescents except in treatment-resistant cases, whereas guidelines for those aged above 18 years are less restrictive [46]. The majority of individuals dispensing antidepressant prescriptions across age categories received their prescriptions from psychiatric specialist services, although there was a more even split between specialist and primary care in the 18–24-year-olds. This mirrors the situation in a German study, where a majority of antidepressant prescriptions were made by child and adolescent psychiatrists in 2011 [10]. However, in a French study, 74.6% of first antidepressant prescriptions to 6- to 17-year-olds were prescribed by general practitioners in 2016 [32]. Across all age groups, the majority of antidepressant users were dispensed antidepressants for more than 1 year, although young adults presented somewhat higher proportions of single dispensed prescriptions than children and adolescents. The proportion of antidepressant users collecting single prescriptions is quite low compared to a study based on German insurance claims data (46.4% of users during 2004–2011) [10]. However, a study of antidepressant prescription in adult populations across five European countries found that the prevalence of SSRI users with one prescription ranged between 13% and 37% in 2008 [42]—a span that encompasses the prevalence of single dispensed prescriptions in young adults in our study. Our study also considers prescriptions dispensed at a pharmacy, a number that is expected to be lower than prescriptions made by a health-care provider. In 2013, 13.5% of antidepressant users dispensed prescriptions for more than one type of antidepressant drug, although this number did not distinguish between concurrent use and “switching” between medication types. A majority of antidepressant prescriptions are part of long-term antidepressant treatment in a number of Western settings [44, 47, 48]. Previous clinical guidelines have mainly focused on treatment initiation and appropriate targeting of antidepressants—research and guidelines on long-term use of antidepressants have been called for [47].

CNS drug polypharmacy with antidepressants increased across age categories during 2006–2013. The youngest group experienced the greatest increase in CNS polypharmacy over the study period, and this was also the age group with the highest proportion of antidepressant users receiving drugs from three or more additional CNS drug classes. A previous study from the USA has found an increase in the prevalence of CNS polypharmacy among 6- to 17-year-old antidepressant users during the period 1996–2007 [21], although more recent studies of this kind are lacking. A trend of increasing CNS polypharmacy warrants attention, as there is a lack of evidence regarding the risk and efficacy of combining CNS drugs in this age group [36]. However, polypharmacy is challenging to address. There is a lack of clarity concerning what is the appropriate treatment of multi-morbidity, and what is overprescription with potentially harmful outcomes [17]. Clinicians often lack direction in this area, as guidelines generally focus on one or a cluster of disorders rather than polypharmacy specifically [49]. Further efforts to improve monitoring of antidepressant use and CNS polypharmacy are necessary [50, 51].

Finally, we also found that antidepressant users were more likely than population controls to use a range of other CNS drugs in 2013. Anxiolytics, hypnotics, and sedatives comprised the most commonly dispensed drug class in conjunction with antidepressants. There were somewhat different co-medication patterns across age groups, partly reflecting distinct proportions of psychiatric diagnoses by age. It is noteworthy that the ORs of receiving other CNS medication concurrently with antidepressants were higher in those under the age of 17 years than in young adults. This may indicate that child and adolescent antidepressant users comprise a more selected clinical group with complex and heterogeneous symptoms that antidepressants alone do not sufficiently address—or that health-care providers mainly prescribe antidepressants in the most severe cases in this age group.

The key strength of this study is that it draws on a nationwide sample of all individuals living in Sweden. This allowed us to assess subgroup-specific patterns in a non-selected sample, giving unique insight into the rise of CNS polypharmacy among young antidepressant users from a complete population cohort. Some limitations should also be considered. First, we used dispensed antidepressant prescriptions as proxies for use, but cannot be sure that the purchased medications were consumed. However, this is closer to the final user than prescription data alone. Second, we did not have information on the indications for antidepressant prescriptions, or on diagnoses that were made in primary care. Finally, these results derive from Sweden and so are not necessarily generalizable to other contexts. While we see stability in demographic patterns of antidepressant prescription across national contexts, further studies are needed to assess whether the clinical patterns are replicated in other countries.

To conclude—in our nationwide sample, we have found several patterns of antidepressant dispensation among children, adolescents, and young adults that require further investigation. The high degree of CNS polypharmacy is a potential cause for concern, as is the rise in antidepressant dispensation generally. These findings highlight the need for studies on the risks and benefits of antidepressants among young users, to inform future research and guideline development.

## Electronic supplementary material

Below is the link to the electronic supplementary material.

Supplementary material 1 (DOCX 29 kb)
