# Supplementary material for: Antidepressant prescription patterns and CNS polypharmacy with antidepressants among children, adolescents, and young adults: a population-based study in Sweden
Source: Eur Child Adolesc Psychiatry. 2019 Jan 19;28(8):1137–45. doi: 10.1007/s00787-018-01269-2 (PMC6675912; doi:10.1007/s00787-018-01269-2)
Supplement: Supplementary file 1 — Supplementary material 1 (DOCX 29 kb) [file 787_2018_1269_MOESM1_ESM.docx]

**SUPPLEMENTARY TABLES**

| **Supplementary table 1. List of antidepressant drugs used in Sweden 2006-2013.** | | | | |
| --- | --- | --- | --- | --- |
| **Selective serotonin reuptake inhibitors (SSRIs)** | **Tricyclic antidepressants (TCAs)** | **Serotonin and norepinephrine reuptake inhibitors (SNRIs)** | **Monoamine oxidase inhibitors (MAOIs)** | **Other antidepressants** |
| N06AB03 Fluoxetine | N06AA02 Imipramine | N06AX16 Venlafaxine | N06AF03 Phenelzine | N06AX02 Tryptophan |
| N06AB04 Citalopram | N06AA04 Clomipramine | N06AX21 Duloxetine | N06AF04 Tranylcypromine | N06AX03 Mianserin |
| N06AB05 Paroxetine | N06AA06 Trimipramine |  | N06AG02 Moclobemide | N06AX11 Mirtazapine |
| N06AB06 Sertraline | N06AA09 Amitriptyline |  |  | N06AX12 Bupropion |
| N06AB08 Fluvoxamine | N06AA10 Nortriptyline |  |  | N06AX18 Reboxetine |
| N06AB10 Escitalopram | N06AA21 Maprotiline |  |  | N06AX22 Agomelatine |

| **Supplementary table 2. Prevalence of antidepressant use in Sweden from 2006 to 2013, by type.** | | | | | | | | | | |
| --- | --- | --- | --- | --- | --- | --- | --- | --- | --- | --- |
|  | **2006** | **2007** | **2008** | **2009** | **2010** | **2011** | **2012** | **2013** | **Relative change (%)^a^** | |
| ***Total no. users*** | 36,462 | 39,324 | 41,422 | 43,532 | 46,395 | 50,578 | 54,679 | 58,854 | 61.4 | |
| ***Type*** |  |  |  |  |  |  |  |  |  |  |
| **TCA^b^** | 0.06 | 0.07 | 0.07 | 0.07 | 0.08 | 0.08 | 0.09 | 0.10 | 52.5 | |
| **SSRI^b^** | 1.1 | 1.2 | 1.2 | 1.3 | 1.4 | 1.5 | 1.6 | 1.7 | 49.5 | |
| **SNRI^b^** | 0.1 | 0.1 | 0.2 | 0.2 | 0.2 | 0.2 | 0.2 | 0.2 | 80.5 | |
| **MAOI^b^** | 0.003 | 0.002 | 0.002 | 0.003 | 0.003 | 0.002 | 0.002 | 0.002 | -24.2 | |
| **Other^b^** | 0.2 | 0.2 | 0.2 | 0.2 | 0.3 | 0.3 | 0.3 | 0.4 | 89.3 | |
| ^a^ Percentage difference between the year 2013 and 2006.  ^b^ Percentage of the total population Swedish population age 6 and above taking the antidepressant subtype;  Tricyclic Antidepressants (TCA); Selective Serotonin Reuptake Inhibitors (SSRI); Serotonin-Norepinephrine Reuptake Inhibitors (SNRI); Monoamine Oxidase Inhibitors (MAOI); Other antidepressants | | | | | | | | | | |

| **Supplementary table 3. The ten most commonly prescribed antidepressant drug types by age category in 2013.** | | | |
| --- | --- | --- | --- |
| **Name** | **ATC** | **Frequency of prescription** | **Percent (within age category)** |
| **Children (0-11 years)** | | | |
| Sertraline | N06AB06 | 9,809 | 62.8 |
| Fluoxetine | N06AB03 | 3,721 | 23.8 |
| Citalopram* | N06AB04 | 603 | 3.9 |
| Amitriptyline | N06AA09 | 475 | 3.0 |
| Mirtazapine* | N06AX11 | 265 | 1.7 |
| Reboxetine* | N06AX18 | 221 | 1.4 |
| Escitalopram* | N06AB10 | 158 | 1.0 |
| Clomipramine* | N06AA04 | 84 | 0.5 |
| Nortriptyline* | N06AA10 | 65 | 0.4 |
| Fluvoxamine | N06AB08 | 50 | 0.3 |
| **Adolescents (12-17 years)** | | | |
| Sertraline | N06AB06 | 115,624 | 49.0 |
| Fluoxetine | N06AB03 | 72,443 | 30.7 |
| Citalopram* | N06AB04 | 16,939 | 7.2 |
| Mirtazapine* | N06AX11 | 10,087 | 4.3 |
| Escitalopram* | N06AB10 | 7,861 | 3.3 |
| Venlafaxine* | N06AX16 | 3,137 | 1.3 |
| Amitriptyline | N06AA09 | 3,133 | 1.3 |
| Bupropion* | N06AX12 | 1,745 | 0.7 |
| Clomipramine* | N06AA04 | 1,304 | 0.6 |
| Duloxetine* | N06AX21 | 1,010 | 0.4 |
| **Young adults (18-24 years)** | | | |
| Sertraline | N06AB06 | 341,143 | 28.6 |
| Citalopram | N06AB04 | 184,861 | 15.5 |
| Fluoxetine | N06AB03 | 157,056 | 13.2 |
| Venlafaxine | N06AX16 | 115,111 | 9.7 |
| Mirtazapine | N06AX11 | 114,534 | 9.6 |
| Escitalopram | N06AB10 | 107,183 | 9.0 |
| Duloxetine | N06AX21 | 45,260 | 3.8 |
| Paroxetine | N06AB05 | 32,647 | 2.7 |
| Amitriptyline | N06AA09 | 27,539 | 2.3 |
| Bupropion | N06AX12 | 23,436 | 2.0 |
| * Not indicated for use in this age group unless at the doctor’s discretion (as per information at fass.se) | | | |

| **Supplementary table 4. Most commonly prescribed anxiolytics, hypnotics, and sedative drugs among antidepressant users in 2013.** | | | |
| --- | --- | --- | --- |
| **Name** | **ATC code** | **Rank in 2013** | **Percentage of total prescriptions of anxiolytics, hypnotics and sedatives** |
| Diazepam | N05BB01 | 1 | 19.0 |
| Oxazepam | N05CF01 | 2 | 18.7 |
| Lorazepam | N05CM06 | 3 | 16.0 |
| Clobazam | N05CH01 | 4 | 15.2 |
| Alprazolam | N05CF02 | 5 | 9.6 |
| Hydroxyzine | N05BA04 | 6 | 8.2 |
| Buspirone | N05BA01 | 7 | 5.7 |
| Chloral hydrate | N05BA12 | 8 | 3.9 |
| Nitrazepam | N05BE01 | 9 | 1.4 |
| Flunitrazepam | N05CD02 | 10 | 0.8 |
| Triazolam | N05CF03 | 11 | 0.4 |
| Midazolam | N05CD03 | 12 | 0.3 |
| Zopiclone | N05BA06 | 13 | 0.2 |
| Zolpidem | N05CM02 | 14 | 0.2 |
| Zaleplon | N05CD08 | 15 | 0.1 |
| Melatonin | N05CD05 | 16 | 0.1 |
| Clomethiazole | N05BA09 | 17 | <0.1 |
| Propiomazine | N05CM09 | 18 | <0.1 |
| Valerianae radix | N05CC01 | 19 | <0.1 |

| **Supplementary Table 5. Diagnoses of common psychiatric diagnoses among antidepressant users in 2013.** | | | | | | | | | |
| --- | --- | --- | --- | --- | --- | --- | --- | --- | --- |
|  |  | **Depression** | **Bipolar disorder** | **Anxiety disorder** | **Schizophrenia spectrum disorder** | **Substance use disorder** | **Personality disorder** | **Attention-deficit/hyperactivity disorder** | **Other developmental or childhood disorder** |
| **Children (Age 0-11)** | **User (N=833)** | 110 (13.2%) | 9 (1.1%) | 326 (39.1%) | 2 (0.2%) | 2 (0.2%) | 2 (0.2%) | 338 (40.6%) | 500 (60.0%) |
|  | **Control (N=833)** | 2 (0.2%) | 0 (0.0%) | 6 (0.7%) | 0 (0.0%) | 2 (0.2%) | 0 (0.0%) | 24 (2.9%) | 64 (7.7%) |
|  | **OR (95% CI)^a^** | 65.6 (16.1, 267.0) |  | 95.6 (42.2, 216.4) |  | 1.0 (0.1, 7.2) |  | 24.0 (15.6, 36.9) | 18.9 (14.1, 25.4) |
| **Adolescents (Age 12-17)** | **User (N=10,033)** | 4,547 (45.3%) | 168 (1.7%) | 5,145 (51.3%) | 112 (1.1%) | 533 (5.3%) | 32 (0.3%) | 2521 (25.1%) | 3,402 (33.9%) |
|  | **Control (N=10,033)** | 102 (1.0%) | 3 (0.0%) | 210 (2.1%) | 2 (0.0%) | 87 (0.9%) | 0 (0.0%) | 316 (3.1%) | 593 (5.9%) |
|  | **OR (95% CI)^a^** | 84.7 (69.4, 103.4) | 57.0 (18.2, 178.6) | 49.7 (43.1, 57.3) | 56.7 (14.0, 229.5) | 6.5 (5.2, 8.2) |  | 10.8 (9.6, 12.2) | 8.9 (8.1, 9.8) |
| **Young adults (Age 18-24)** | **User (N=47,988)** | 19,074 (39.7%) | 2,060 (4.3%) | 21,825 (45.5%) | 1,194 (2.5%) | 7,151 (14.9%) | 2,872 (6.0%) | 6,838 (14.2%) | 7,270 (15.1%) |
|  | **Control (N=47,988)** | 1,372 (2.9%) | 184 (0.4%) | 2,312 (4.8%) | 134 (0.3%) | 1,682 (3.5%) | 163 (0.3%) | 1,202 (2.5%) | 1,485 (3.1%) |
|  | **OR (95% CI)^a^** | 22.5 (21.3, 23.8) | 11.7 (10.01, 13.6) | 16.6 (15.8, 17.4) | 9.2 (7.7, 11.0) | 4.9 (4.6, 5.1) | 19.0 (16.2, 22.3) | 6.6 (6.2, 67.0) | 5.7 (5.4, 6.1) |
| ^a^ Adjusted for sex and age (in one-year age bands). | | | | | | | | | |

**Supplementary table 6. Marketing authorizations/indications of the most common antidepressant drugs in children, adolescents, and young adults (source: www.fass.se).**

|  |  | **Indication/authorization by age group^a,b^** | | |
| --- | --- | --- | --- | --- |
| **Drug** | **Antidepressant type** | **Children**  **(0-11 years)** | **Adolescents**  **(12-17 years)** | **Young adults**  **(18-24 years)** |
| Amitriptyline | TCA | Enuresis (from 6 years) | Enuresis | Depression; Neuropathic pain; Prophylactic treatment of chronic tension headache; Prophylactic treatment of migraine |
| Bupropion | Other |  |  | Depression |
| Citalopram | SSRI |  |  | Severe depression; Panic disorder; OCD |
| Clomipramine | TCA |  |  | Depression; OCD; Social phobia; Panic disorder; Narcolepsy |
| Duloxetine | SNRI |  |  | Depression; Generalized anxiety disorder; Painful diabetic neuropathy |
| Escitalopram | SSRI |  |  | Depression; Anxiety disorders; Panic disorder; OCD |
| Fluoxetine | SSRI | Medium to severe depression (from 8 years), if no results from 4-6 sessions of psychological treatment | Medium to severe depression, if no results from 4-6 sessions of psychological treatment | Depression; OCD; Bulimia |
| Fluvoxamine | SSRI | OCD (from 8 years) | OCD | Depression; OCD |
| Mirtazapine | Other |  |  | Depression |
| Nortriptyline | TCA |  |  | Depression |
| Paroxetine | SSRI |  |  | Depression; OCD; Panic disorder; Generalized anxiety disorder; Social phobia; PTSD |
| Reboxetine | Other |  |  | Depression (acute treatment) |
| Sertraline | SSRI | OCD (from 6 years) | OCD | Depression; Social phobia; PTSD; Panic disorder; OCD |
| Venlafaxine | SNRI |  |  | Depression; Generalized anxiety disorder; Social phobia; Panic disorder |
| ^a^ OCD: “Obsessive Compulsive Disorder”; PTSD: “Post-Traumatic Stress Disorder”  ^b^ All information on indications derives from Pharmaceutical Specialties in Sweden (“Farmaceutiska specialiteter i Sverige”; [www.fass.se](http://www.fass.se)), produced by LIF (The Swedish Association of The Pharmaceutical Industry; “Läkemedelsindustriföreningen”), the trade association for the Swedish research-based pharmaceutical industry. | | | | |
